# Long-Term Visual Quality and Pupil Changes after Small-Incision Lenticule Extraction for Eyes without Preoperative Cylinder Refraction

**DOI:** 10.1155/2024/8835585

**Published:** 2024-01-20

**Authors:** Xiaonan Yang, Qiting Feng, Quan Liu, Jianhui Chen, Pengxia Wan

**Affiliations:** ^1^Department of Ophthalmology, The First Affiliated Hospital, Sun Yat-sen University, Guangzhou 510080, Guangdong, China; ^2^State Key Laboratory of Ophthalmology, Zhongshan Ophthalmic Center, Sun Yat-Sen University, Guangzhou, China

## Abstract

**Purpose:**

To investigate the long-term changes in visual quality and pupil size after small incision lenticule extraction (SMILE) for eyes without preoperative cylinder refraction.

**Methods:**

Thirty-three myopic eyes (33 patients) without preoperative cylinder refraction were corrected using SMILE. Refractive outcomes, corneal curvature, aberrations, contrast sensitivity (CS), and pupil diameter were evaluated preoperatively, and 30 months postoperatively.

**Results:**

The 30-month postoperative uncorrected and corrected distance visual acuity (UDVA and CDVA, LogMAR) were −0.10 ± 0.09 and −0.14 ± 0.06, respectively, whereas the preoperative CDVA (LogMAR) was −0.07 ± 0.05. Cylinder refraction of −0.11 ± 0.21 D (ranging from −0.50 to 0.00) was observed at 30 months postoperatively, increasing from the preoperative cylinder refraction of 0.00 ± 0.00 D (*P*=0.004). Moreover, the centroid coordinates *x*, *y* of corneal anterior astigmatic vectors were −0.19 ± 0.22, 0.81 ± 0.33 at 30 months postoperatively, and 0.02 ± 0.28, 0.76 ± 0.51 preoperatively (*P*_*x*_ < 0.001 and *P*_*y*_=0.810, respectively). Furthermore, a 15° axis change in the mean anterior corneal astigmatic vector was observed at 30 months postoperatively from the preoperative state, as measured by Pentacam. At 30 months postoperatively, the photopic Log CS reduced significantly with glare at three and six cycles/degrees (*P* < 0.001 and *P*=0.015, respectively), a decreased photopic pupil diameter (3.27 ± 0.55 mm vs. 3.10 ± 0.66 mm, *P*=0.030), and an increased Coma (*Z*_3_^1^) and Trefoil (*Z*_3_^−3^) at 4 mm diameter area analysis. However, a significant linear regression relationship was only observed between changes in photopic pupil diameter and changes in photopic Log CS with glare at 12 cycles/degree (*P*=0.038 and *β* = 0.282).

**Conclusion:**

Slight cylinder regression was observed with thicker corneal lenticular extraction after SMILE correction of nonastigmatic eyes 30 months postoperatively. This regression was mainly because of the axis changes in anterior corneal astigmatism power. Therefore, a cylinder nomogram modification of 0.25 to 0.50 D is considerable for correcting nonastigmatic myopic eyes with a predicted spherical lenticular thickness over 100 *µ*m.

## 1. Introduction

Refractive surgery works by correcting the spherical and cylindrical errors on the corneal plane of the patient's eye using a laser [1–3]. In most cases, corneal laser surgery aims to achieve emmetropia by neutralizing the entire spherocylindrical error. Any discrepancy between the desired correction and the actual surgical outcome can cause unexpected residual refractive errors. Spherical ablation with an excimer laser is designed to flatten all meridians, correcting myopia without changing the magnitude or meridian of astigmatism. However, induced excessive astigmatism has been reported after photorefractive keratectomy (PRK) and laser in situ keratomileusis (LASIK), possibly owing to spatially heterogeneous tomographic healing [1, 4–7]. To mitigate this, the design of refractive excimer lasers has improved featuring higher repetition rates and smaller laser spot sizes [8, 9]. However, the excimer laser induces more corneal healing processes, such as keratocyte apoptosis, proliferation, and inflammation, than the femtosecond laser, representing a great advancement in laser surgery [10].

Small incision lenticule extraction (SMILE) is a newly developed “flapless” and all-in-one surgical method performed using the VisuMax® femtosecond laser (Carl Zeiss Meditec AG, Jena, Germany). Laser energy triggers optical breakdown, known as photo-disruption, in the cornea and generates plasma, resulting in the severing of the tissue in the region of interest. In SMILE, visible structural alterations in the surrounding tissue or surface of the cornea are minimal. The laser has a high repetition rate of 500 kHz and a spot diameter of less than 3 *µ*m. Theoretically, the inner surface of the cap should be parallel to the residual stromal surface, particularly with spherical ablation. Spherical correction via SMILE surgery alone should not induce excessive astigmatism. However, the clinical data supporting this hypothesis are lacking. Thus, this study aimed to determine whether the spherical ablation during SMILE induces long-term astigmatism.

## 2. Materials and Methods

### 2.1. Patient Selection

This prospective study enrolled 33 patients (33 right eyes). SMILE procedures were conducted at the refractive suite of the Zhongshan Ophthalmic Center, Sun Yat-sen University (Guangzhou, People's Republic of China) between June 2015 and February 2016. Inclusion criteria involved patients with a minimum age of 18 years, stable myopia for at least 1 year, myopia without cylinder refraction (cylinder refraction = 0 D), emmetropia as the attempted correction (excluding eyes targeted for monovision), and a residual stromal thickness exceeding 250 *µ*m. Patients with a history of keratoconus, corneal lesions, corneal surgery, severe cataracts, glaucoma, or posterior abnormalities (including choroidal neovascularization, retinoschisis, retinal detachment, or macular holes) were excluded. Patients were thoroughly briefed on SMILE surgery, including its potential side effects and complications. Informed consent was obtained from all participants using a consent form approved by the Research Ethics Board of the Zhongshan Ophthalmic Center (Certificate no. 2013MEKY036). This study was conducted following the tenets of the Declaration of Helsinki.

### 2.2. SMILE Procedure

A highly experienced surgeon (Q. L.) performed all SMILE procedures to avoid operative errors. The intended lenticular diameter (optical zone) was set at 6.5 to 7.1 mm to maintain a safe residual bed thickness and minimize the risk of postoperative glare. Cap diameter varied from 7.5 to 7.9 mm, with an intended cap thickness ranging from 120 to 130 *µ*m. An additional of −0.25 D spherical correction was used for patients under 40 years.

### 2.3. Examinations

Manifest refraction was measured preoperatively and 1 week, 1 month, 3 months, 6 months, and 30 months post-operatively. The uncorrected and corrected distance visual acuities (UDVA and CDVA) were measured using a Snellen chart. The safety index was calculated by dividing the 30-month postoperative CDVA (in decimals) by the preoperative CDVA (in decimals). This index represents the safety of SMILE from a CDVA perspective. A safety index >1.0 indicated satisfactory safety of SMILE. The efficacy index was calculated by dividing the 30-month postoperative UDVA (in decimals) by the preoperative CDVA (in decimals). The efficacy of SMILE was evaluated based on UDVA. An efficacy index >1.0 indicated satisfactory efficacy of SMILE. The attempted spherical equivalent (SE) is the planned SE corrected using refractive surgery, and the achieved SE was the actual SE corrected using SMILE, calculated by subtracting the postoperative SE from the preoperative SE. Refractive predictability was assessed by the percentage of eyes with <0.5 D of target correction using the SE. SE stability was assessed by comparing the postoperative cycloplegic refractions SE at follow-up times. The magnitude of the surgically induced astigmatism (SIA) vector was evaluated 30 months postoperatively. Cylinder refraction stability was assessed by comparing the postoperative cylinder refraction at different follow-up times.

The Oculyzer (Oculus Optikegerate Gmbh, Wetzlar, Germany) was used to measure the photopic pupil diameter and anterior corneal astigmatic power preoperatively and 30 months postoperatively. Only scans graded as “OK” using the instrument were included for further analyses. A vector comparison of anterior corneal astigmatism power between the 30-month postoperative and preoperative period was performed. Similar to the study by Holladay [11], we used an axis form to describe the direction of the power notation.

The wavefront supported custom ablation (WASCA) aberrometer (Carl Zeiss Meditec, Jena, Germany) was used to measure the ocular aberrations, including ocular total high-order aberration (HOA) root-mean-square (computed for third to sixth Zernike terms), primary coma (the Zernike terms *Z*_3_^±1^), trefoil (the Zernike terms *Z*_3_^±3^), secondary astigmatism (the Zernike terms *Z*_4_^±2^), tetrafoil (the Zernike terms *Z*_4_^±4^), and primary spherical aberration (the Zernike terms *Z*_4_^0^) preoperatively, 6 months and 30 months after surgery, under 0.1 cd/m^2^ scotopic light settings. Only monocular testing was conducted using the WASCA. High ocular aberrations were analyzed for a 4.0 mm zone (excluding eyes with scotopic pupil diameter less than 4.0 mm), a 6.0 mm zone (excluding eyes with scotopic pupil diameter less than 6.0 mm), and a scotopic pupil diameter zone. Natural scotopic pupil diameter was recorded preoperatively and 30 months postoperatively. Contrast sensitivity (CS) was measured at a 2.5 m distance under CDVA and four lighting conditions (Vector Vision CSV 1000E test; Vector Vision, City, USA). These included photopic CS with or without glare, and mesopic CS with or without glare at spatial frequencies of 3 (A), 6 (B), 12 (C), and 18 (D) cycles/degrees.

### 2.4. Statistical Analyses

Only one eye of each patient was included in the statistical analyses. All data were collected and recorded using Excel 2016 (Microsoft, Inc., Redmond, WA, USA). A standardized nine graph format was used to present refractive surgery results. All statistical analyses were performed using SPSS statistical analyses software v22.0 (SPSS Inc., Chicago, USA). Visual acuity was converted to LogMAR for statistical analyses. Quantitative variables are expressed as mean ± standard deviation. Repeated measures of a general linear model were used for multiple comparisons of preoperative and postoperative quantitative results. A linear regression model was used to analyze the relationship between changes in photopic pupil size and changes in log CS. Moreover, the involved eyes were divided into two groups for further analysis: eyes without postoperative cylinder regression at 30 months and those with postoperative cylinder regression of at least 0.25 D. Statistical significance was set at a *P* value of <0.05.

## 3. Results

In total, 33 eyes from 33 patients, with a mean age of 24.87 ± 5.52 years (ranging from 18 to 39 years), underwent SMILE. The SE was equal to the preoperative myopic spherical refraction which was −4.24 ± 1.40 D.  Table 1 summarizes the baseline eye characteristics.

### 3.1. Efficacy Index Analysis

**Figure 1:**
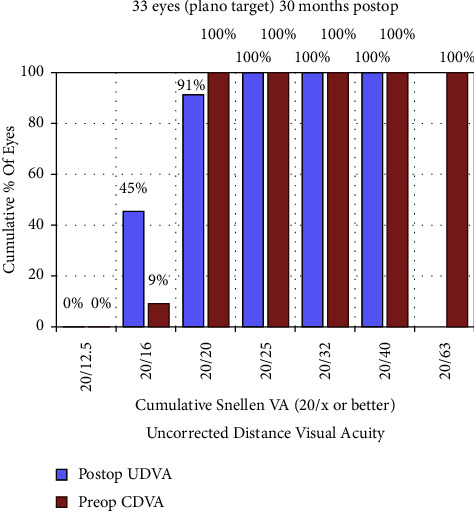
Cumulative percentage of eyes attaining specified cumulative levels of uncorrected distance visual acuity (UDVA) 30 months after surgery (all eyes had emmetropia as the target refraction). CDVA = corrected distance visual acuity.

**Figure 2:**
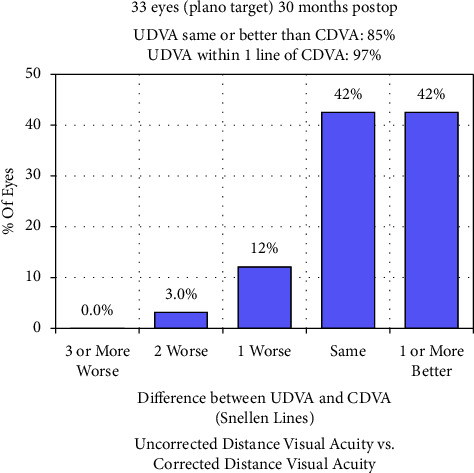
Percentage of eyes comparing 30 months postoperative uncorrected distance visual acuity (UDVA) and preoperative corrected distance visual acuity (CDVA).

Ninety-one percent of the eyes had a postoperative UDVA of 20/20 or more (Figure 1). As shown in Figure 2, 85% of the eyes had a postoperative UDVA equal to or better than the preoperative CDVA. Additionally, 12% had a one-line decrease in UDVA than the preoperative CDVA, 3% had a two-line reduction, and no eyes had a decrease of three or more lines. The efficacy index was 1.09 ± 0.21. Furthermore, compared with the preoperative CDVA (LogMAR −0.07 ± 0.05), the postoperative UDVA was better at 1 week (−0.12 ± 0.06, *P* < 0.001), 1 month (−0.14 ± 0.07, *P* < 0.001), 3 months (−0.15 ± 0.06, *P* < 0.001), 6 months (−0.14 ± 0.05, *P* < 0.001), and 12 months (−0.14 ± 0.05, *P* < 0.001). No significant improvement was observed between the 30-month postoperative UDVA (−0.10 ± 0.09) and preoperative CDVA (*P* = 0.083).

### 3.2. Safety Index Analysis

**Figure 3:**
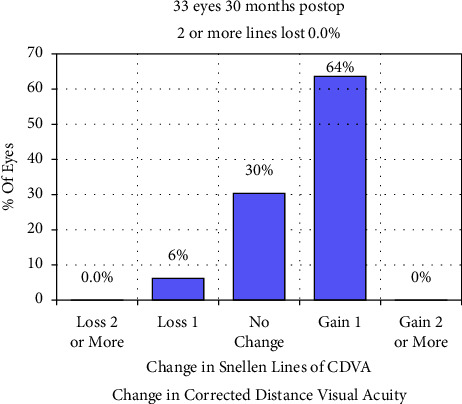
Gain and loss of corrected distance visual acuity (CDVA) 30 months postoperatively.

Postoperative CDVA (LogMAR) significantly improved more than preoperative CDVA (Table 2). Specifically, 30% of the eyes exhibited no change in CDVA, 64% gained one line, and 6% lost one line (Figure 3). The safety index was 1.20 ± 0.18. All surgeries were completed without intraoperative or postoperative complications. No cases of keratectasia were observed during the follow-up.

### 3.3. Predictability Analysis

**Figure 4:**
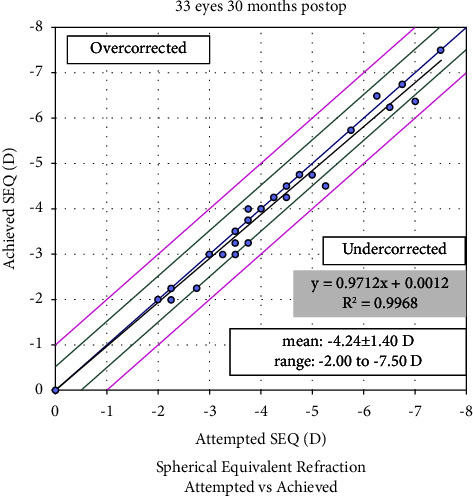
Attempted spherical equivalent (SEQ) refractive change plotted against the achieved SEQ refractive change at 30 months.

**Figure 5:**
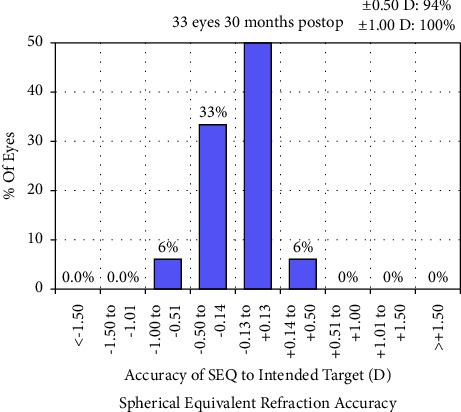
Percentage of eyes attaining specified differences in attempted versus achieved correction at 30 months. SEQ, spherical equivalent.

Figure 4 displays a scatterplot of attempted versus achieved SE correction 30 months postoperatively. At 30 months postoperatively, 94% of the eyes were within ±0.50 D, and 100% were within ±1.0 D of the attempted correction (Figure 5).

### 3.4. Stability Analysis

**Figure 6:**
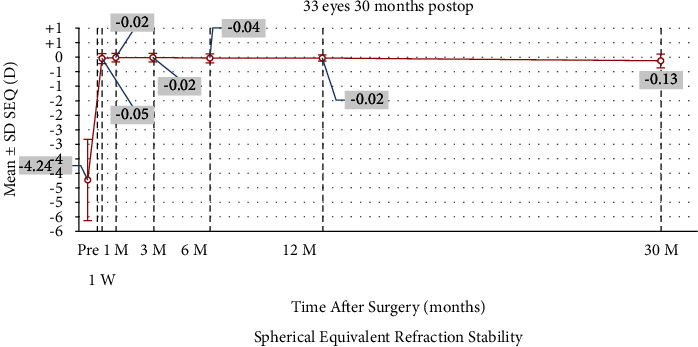
Stability of manifest spherical equivalent after small incision lenticule extraction over the 30-month follow-up period. SEQ, spherical equivalent.

Figure 6 reveals that postoperative SE refraction fluctuated from −0.05 D at 1 week to −0.13 D at 30 months. However, no significant changes were observed in postoperative SE, sphere refraction, or cylinder refraction from 1 week to 30 months (Table 2).

### 3.5. Refractive Astigmatism Analysis

**Figure 7:**
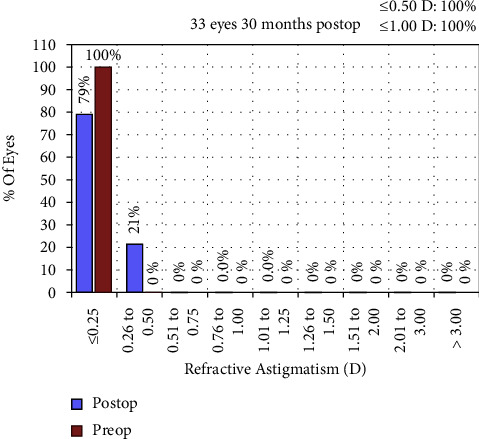
Cumulative percentage of eyes attaining specified cumulative levels of refractive astigmatism 30 months after surgery (all eyes had emmetropia as the target refraction).

**Figure 8:**
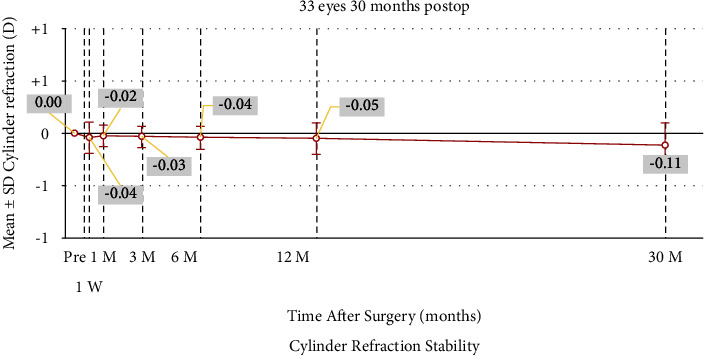
Stability of manifest cylinder refraction after small incision lenticule extraction over the 30-month follow-up period. SEQ, spherical equivalent.

**Figure 9:**
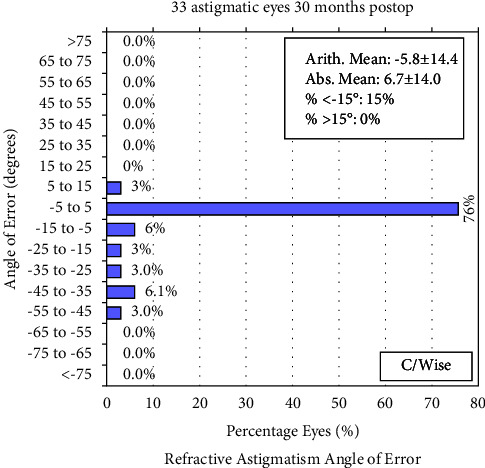
Percentage of eyes according to the angle of error (degrees) after small incision lenticule extraction at 30 months.

Figures 7–9 depict the refractive astigmatism results. As shown in Table 2, postoperative cylinder refraction was −0.11 ± 0.21 D at 30 months, which was significantly different from the preoperative state (*P*=0.004). Specifically, 3.0% of the eyes had a cylinder refraction ranging from 0.25 D to 0.50 D at 30 months postoperatively, which was 12.0% at 6 months. The percentage of eyes with cylinder refraction ranging from 0.50 D to 0.75 D was 21.2%, whereas it was 0.0% at 6 months; no eye had a cylinder more than 0.75 D after 6 months postoperatively (Table 3). The mean vector of the 30-month postoperative cylinder refraction was −0.08 × 35.5°.

At 30 months post-operatively, eight (24.2%) eyes had a cylinder refraction of no less than 0.25 D.  Table 4 presents a comparison of preoperative parameters between two groups: group one included 30-month postoperative nonastigmatic eyes with zero-cylinder refraction (25 eyes) and group two included the postoperative astigmatic group with cylinder refraction above zero (8 eyes). Only predicted lenticular thickness showed a significant difference between these two groups (*P*=0.018).

### 3.6. Changes in Anterior Corneal Astigmatic Vector

**Figure 10:**
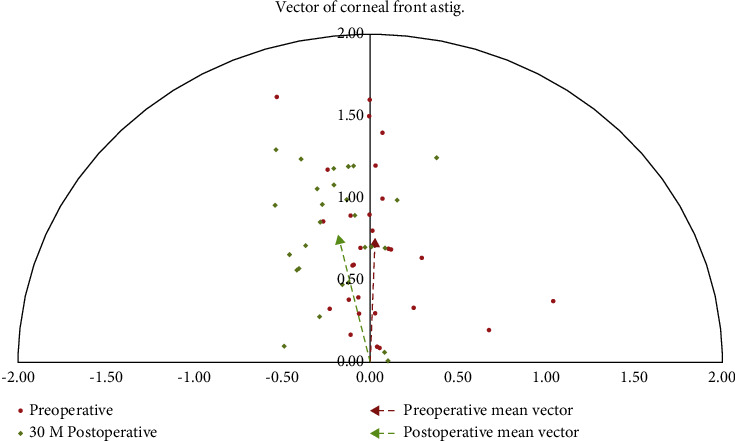
Fifteen degrees axis change of the mean anterior corneal astigmatic vector was observed at 30 months postoperatively from the preoperative state measured by Pentacam.

The magnitude of the anterior corneal astigmatic power was 0.85 ± 0.36 D at 30 months postoperatively, whereas it was 0.84 ± 0.51 D preoperatively. No significant difference in the magnitude of corneal astigmatism power was observed between the preoperative and 30-month postoperative measurements (*P*=0.926). However, the centroid coordinates *x*, *y* of corneal anterior astigmatic power were −0.19 ± 0.22 and 0.81 ± 0.33 at 30 months postoperatively, whereas it was 0.02 ± 0.28 and 0.76 ± 0.51 preoperatively. A significant difference was observed postoperatively in the *x*-centroid coordinates (*P* < 0.001), whereas no significant difference was observed in the *y*-centroid coordinates (*P*=0.810) postoperatively, compared with the preoperative coordinates. Furthermore, the mean astigmatic power in vector form was 0.76 D × 178.3° preoperatively and 0.80 D × 13.3° at 30 months postoperatively. The mean angle error of the anterior corneal astigmatism power in the vector form was negative 15° at 30 months compared with the preoperative state (Figure 10).

### 3.7. Changes in Ocular Aberrations

**Figure 11:**
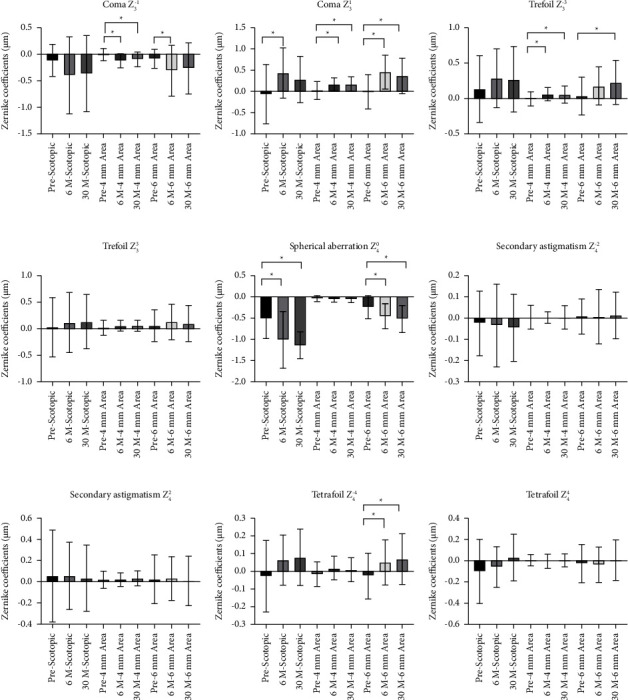
Comparison of ocular aberrations pre- and postoperatively. Only the *P* values <0.05 were noted in the figure with “^*∗*^”.

The pre-and postoperative ocular aberrations were analyzed at different diameters, including 4.0 mm, 6.0 mm, and natural scotopic pupil diameter (Figure 11 and Table 5).

### 3.8. CS Analysis

Table 6 shows the CS under photopic and scotopic conditions, with and without glare, at the four spatial frequencies. The photopic and scotopic Log CS remained unchanged from preoperative to postoperative up to the 30-month follow-up (*P* > 0.05). However, the Log CS under the photopic glare conditions reduced at three (*P* < 0.001) and six (*P*=0.015) cycles/degrees at 30 months postoperatively.

### 3.9. Changes in Pupil Diameters

The photopic pupil diameter, measured using the Oculyzer, significantly decreased at 30 months postoperatively (3.27 ± 0.55 mm vs. 3.10 ± 0.66 mm, *P*=0.030, Table 7). No significant change was observed in the scotopic pupil diameter, measured using WASCA, between the preoperative and 30-month postoperative (6.67 ± 0.52 mm vs. 6.70 ± 0.78 mm, *P*=0.889, Table 7).

Furthermore, a significant linear regression relationship was observed only between changes in photopic pupil diameter and changes in photopic Log CS with glare at 12 cycles/degrees (Table 8).

## 4. Discussion

To our knowledge, this study is the first to evaluate SIA using SMILE in nonastigmatic eyes. Previous research has mainly focused on clinical outcomes of SMILE procedures for correcting myopic eyes with preoperative low, moderate, and high cylinder refraction [12, 13]. The hypothesis that long-term wound healing would induce cylinder refraction with SMILE surgeries remains unverified. However, our study evaluated eyes with myopia without preoperative cylinder. Traditionally, the long-term efficacy, safety, predictability, and stability of these procedures have been evaluated. Objective and subjective visual quality and long-term changes in pupil diameter under photopic and scotopic conditions were determined. We investigated potential factors inducing SIA in SMILE procedures, revealing that thicker lenticules (over 100 *µ*m) extraction may heighten the risk of SIA.

Our results concerning the long-term efficacy, safety, predictability, and stability of SMILE surgery, suggest that nonastigmatic myopic eyes might benefit more than astigmatic ones from SMILE procedures, even after 30 months [12, 14–19]. Specifically, the efficacy index, at 1.09 ± 0.21, higher than 1.00, implied that most patients achieved better postoperative UDVA than preoperative CDVA, indicating a better visual experience than wearing glasses. Furthermore, no significant regression was observed in SE or spherical refraction, which was slightly different from the findings from previous studies [15, 17, 19–21]. Higher myopia or cylinder refraction might pose a greater risk of experiencing significant regression. Moreover, a minor augmentation in the procedure might be essential for correcting moderate myopic eyes without astigmatism, such as −0.25 D or −0.50 D, causing 30-month postoperative SE at −0.13 ± 0.23 D [22, 23]. At the 30-month time point, the SE was −0.13 ± 0.23 D relative to −4.24 ± 1.40 D preoperatively. Only 6% of the eyes exhibited an unexpected SE within −0.50 to −1.00 D range, whereas 94% had the expected postoperative SE (Figure 5). However, the 30-month postoperative UDVA (Log MAR) of −0.10 ± 0.09 closely approximated the preoperative CDVA of −0.07 ± 0.05 (*P* > 0.999). Consequently, an increment in −0.25 D sphere refraction may be useful for moderate myopia correction. However, a −0.25 D added sphere refraction was conservative compared with the recommended magnitude of 10% of the preoperative refractive error [13, 22].

A slight cylinder refraction of −0.11 D was observed in our study at 30 months postoperatively. Moreover, thicker corneal lenticules were more likely to induce SIA, especially when lenticular thickness surpassed 95.88 *µ*m. The percentage of eyes with SIA was higher at 30 months (24%) than at 6 months (12%). Furthermore, significant rotation was observed in the anterior corneal astigmatic power at 30 months postoperatively versus preoperatively (Figure 10). The plastic action of the eyelid on the cornea may play a role in postoperative cylinder refraction. In our study, the incision was performed at the 11 o'clock position, but not at the 12 o'clock position, as some researchers recommended, causing a different rotation in the front corneal astigmatism power vector [7]. No significant refractive cylinder changes were observed from the preoperative to the 12-month postoperative period. These findings suggest that the uncertainty of asymmetrical healing patterns reported in PRK or LASIK and the device-related errors might not be attributed to the significant postoperative cylinder changes in SMILE. In Karmona et al.'s study [1], the postoperative cylinder was −0.36 ± 0.4 D 3 months after spherical myopic LASIK. Another study observed that the postoperative cylinder fluctuated from −0.66 ± 0.29 D at 1 month to −0.49 ± 0.34 D at 6 months [4]. The induced astigmatic refraction after LASIK was higher than those obtained from our results. The reasons for induced astigmatism after LASIK or PRK might be the underlying stromal irregularities, unequal flap retraction, and compensatory epithelial hyperplasia, particularly in higher myopia [24]. Hence, SMILE may be superior to LASIK and PRK in treating nonastigmatic myopic eyes because of its flapless nature and reduced damage response. However, a cylinder nomogram modification of 0.25 to 0.50 D of SMILE procedures is still considerable for correcting nonastigmatic myopic eyes with a predicted spherical lenticular thickness over 100 *µ*m.

The reduced 30-month postoperative Log CS from the preoperative state at low spatial frequencies under photopia with glare conditions was observed in our study (Table 3). Xu et al. have suggested that small pupils significantly reduced best-focus vision under low photopic conditions by having optical aberrations and diffraction that attenuate retinal image quality [25]. However, the aforementioned study highlighted the diffractive or smaller pupil sizes (less than 2 mm), which reduces image quality due to additional diffraction from a very small pupillary edge. Generally, a smaller pupil size should reduce aberrations and increase the depth of the field, improving image quality. In this study, the postoperative photopic pupil diameter was 3.10 ± 0.66 mm, ranging from 2.32 mm to 4.48 mm. Consequently, changes in the pupil diameter here could not explain the reduced long-term CS under photopia with glare conditions. But, both Coma (*Z*_3_^1^) and Trefoil (*Z*_3_^−3^) significantly increased at 4 mm analyzed zone 30 months postoperatively, which would affect the CS quality under photopic conditions. Objective and subjective night vision quality were evaluated in our study. At 30 months postoperatively, ocular HOAs at a 6.0 mm diameter and at a scotopic pupil diameter of approximately 6.70 mm exhibited a significant increase postoperatively versus the preoperative including total HOAs, coma, and spherical aberration. However, no significant change was observed in CS under scotopic conditions with or without glare. The increased HOAs, including coma and spherical aberration, were observed at 6.0 mm diameter and scotopic diameter of approximately 6.7 mm, possibly owing to a functional optical zone of approximately 4.0 mm after SMILE [17, 19, 26, 27]. In our research, even with the increased HOAs under scotopic diameter, no significant change was observed in night objective visual quality, displayed by CS under scotopic conditions with or without glare. CS measurements might not be sufficiently sensitive to evaluate the patients' night visual quality. Therefore, more objective and sensitive measures of nighttime visual quality are required.

This study had some limitations. First, the sample size was small: fortunately, this defect did not affect our conclusion. Second, this study was limited by the absence of control groups for LASIK and PRK. A comparison of the clinical outcomes between SMILE and LASIK or PRK for correcting nonastigmatic myopic eyes would better demonstrate the advantages of SMILE. Third, a new method for testing objective night visual quality must be developed.

## 5. Conclusions

This study is the first article to investigate the long-term SIA after SMILE for nonastigmatic myopic eyes. This finding reveals that at 30 months postoperatively, more induced cylinder refraction was observed with thicker corneal ablation, which might result from changes in the axis of the anterior corneal astigmatism power. Additionally, decreased CS under photopic conditions with glare may be due to the increased HOAs at the 4 mm zone.

## Figures and Tables

**Table 1:** Preoperative patient demographics of the eyes undergoing SMILE.

	Mean ± SD
Age (years)	24.87 ± 5.52 (18 to 39)
Male-to-female ratio	0.83
Sphere refraction (D)	−4.24 ± 1.40 (−7.5 to −2)
Central corneal thickness (*µ*m)	544.3 ± 21.4 (509 to 592)
Corrected distance visual acuity (LogMAR)	−0.07 ± 0.05 (−0.18 to 0.00)

**Table 2:** Visual acuity and manifest refraction of the study eyes.

	Preop	1 week	1 month	3 month	6 month	12 month	30 month
CDVA (LogMAR)	−0.07 ± 0.05	−0.14 ± 0.06	−0.16 ± 0.05	−0.16 ± 0.05	−0.16 ± 0.04	−0.14 ± 0.05	−0.14 ± 0.06
		*p*_1w‐before<0.001_	*p*_1m‐before<0.001_	*P*_3m‐before<0.001_	*P*_6m‐before<0.001_	*P*_12m‐before<0.001_	*P*_30m‐before<0.001_

Sphere (D)	−4.24 ± 1.40	−0.03 ± 0.20	−0.01 ± 0.12	0.00 ± 0.14	−0.02 ± 0.14	0.01 ± 0.10	−0.08 ± 0.19
		*p*_1w‐before<0.001_	*p*_1m‐before<0.001_	*P*_3m‐before<0.001_	*P*_6m‐before<0.001_	*P*_12m‐before<0.001_	*P*_30m‐before<0.001_

Cylinder (D)	0.00 ± 0.00	−0.04 ± 0.15	−0.02 ± 0.10	−0.03 ± 0.10	−0.04 ± 0.11	−0.05 ± 0.15	−0.11 ± 0.21
							*p*_30m‐before_=0.004

Spherical equivalent (D)	−4.24 ± 1.40	−0.05 ± 0.17	−0.02 ± 0.15	−0.02 ± 0.16	−0.04 ± 0.16	−0.02 ± 0.11	−0.13 ± 0.23
		*p*_1w‐before<0.001_	*p*_1m‐before<0.001_	*P*_3m‐before<0.001_	*P*_6m‐before<0.001_	*P*_12m‐before<0.001_	*P*_30m‐before<0.001_

Repeated measures of the general linear model were applied. Only *P* value <0.05 is shown in the table. CDVA: corrected distance visual acuity, UDVA: uncorrected distance visual acuity, SE: spherical equivalent, and Preop: preoperative.

**Table 3:** Percentage of nonastigmatic patients with induced astigmatism after SMILE.

SIA (D)	6-month (%)	30-month (%)
≥0.25	12.0	3.0
≥0.50	0.0	21.2
≥0.75	0.0	0.0
≥1.00	0.0	0.0

*Note*. SIA: surgically induced astigmatism.

**Table 4:** The preoperative clinical parameter comparison between group one and group two.

	Group one	Group two	*P* value
	(*n* = 25)	(*n* = 8)	
Pre. sphere (D)	−4.07 ± 1.39	−4.78 ± 1.38	0.215
Male-to-female ratio	1.27	0.33	0.131
Pre. central corneal thickness (*µ*m)	540.95 ± 20.83	546.13 ± 26.48	0.591
Cap thickness (*µ*m)	129.47 ± 2.29	127.50 ± 4.63	0.147
Cap diameter (*µ*m)	7.63 ± 0.16	7.65 ± 0.21	0.801
Lenticular thickness (*µ*m)	78.05 ± 15.36	95.88 ± 19.78	*0.018* ^*∗*^
Pre. *K*_*m*_ (D)	43.03 ± 1.44	43.96 ± 1.46	0.135
Pre. astig. power (D)	−0.78 ± 0.52	−0.98 ± 0.48	0.385

*Note*. ^*∗*^*P* < 0.05. Pre.: preoperative; *K*_*m*_: mean keratometry of cornea; astig.: astigmatism. Group one means the group involved eyes without postoperative cylinder regression at 30 months, which also means that in this group, both the preoperative and the 30-month postoperative cylinder refraction was zero. Group two means the group involved the other eyes having postoperative cylinder regression no less than 0.25 D, which also means that in this group, the preoperative cylinder refraction was zero, while the postoperative cylinder refraction was ≥0.25 D or ≤−0.25 D.

**Table 5:** High ocular aberrations preoperative and postoperatively.

		Preoperative	6-month	30-month
tHOA (RMS, *μ*m)	Scotopic	0.43 ± 0.18	0.65 ± 0.18	0.64 ± 0.14
			*P*_6m‐before<0.001_	*P*_30m‐before<0.001_
	4 mm	0.10 ± 0.06	0.12 ± 0.04	0.13 ± 0.04
	6 mm	0.27 ± 0.10	0.42 ± 0.11	0.43 ± 0.12
			*P*_6m‐before<0.001_	*P*_30m‐before<0.001_

Coma (*Z*_3_^−1^, *μ*m)	Scotopic	−0.11 ± 0.30	−0.40 ± 0.72	−0.36 ± 0.71
	4 mm	−0.01 ± 0.11	−0.12 ± 0.13	−009 ± 0.14
	6 mm	−0.09 ± 0.18	−0.31 ± 0.48	−0.27 ± 0.48
			*P*_6m−before=0.030_	

Coma (*Z*_3_^1^, *μ*m)	Scotopic	−0.06 ± 0.70	0.43 ± 0.59	0.28 ± 0.54
			*P*_6m‐before=0.013_	
	4 mm	0.03 ± 0.21	0.17 ± 0.15	0.18 ± 0.17
			*P*_6m‐before=0.005_	*P*_30m‐before=0.006_
	6 mm	−0.01 ± 0.47	0.46 ± 0.40	0.37 ± 0.42
			*P*_6m‐before<0.001_	*P*_30m‐before=0.002_

Trefoil (*Z*_3_^−3^, *μ*m)	Scotopic	0.13 ± 0.47	0.29 ± 0.42	0.27 ± 0.46
	4 mm	−0.01 ± 0.10	0.06 ± 0.10	0.06 ± 0.12
			*P*_6m‐before=0.017_	*P*_30m‐before=0.048_
	6 mm	0.04 ± 0.27	0.18 ± 0.27	0.23 ± 0.31
				*P*_30m‐before=0.022_

SA (*Z*_4_^0^, *μ*m)	Scotopic	0.50 ± 0.48	−1.01 ± 0.67	−1.15 ± 0.31
			*P*_6m‐before=0.005_	*P*_30m‐before<0.001_
	4 mm	−0.04 ± 0.07	−0.06 ± 0.06	−0.07 ± 0.07
	6 mm	−0.24 ± 0.28	−0.46 ± 0.29	−0.52 ± 0.32
			*P*_6m‐before=0.010_	*P*_30m‐before=0.002_

Tetrafoil (*Z*_4_^−4^, *μ*m)	Scotopic	−0.02 ± 0.20	0.07 ± 0.14	0.08 ± 0.16
	4 mm	−0.02 ± 0.07	0.02 ± 0.07	0.01 ± 0.07
	6 mm	−0.02 ± 0.13	0.05 ± 0.13	0.07 ± 0.14
			*P*_6m‐before=0.035_	*P*_30m‐before=0.015_

“Scotopic” in this table means scotopic pupil diameter measured by WASCA. Only *P* value <0.05 is shown in the table and indicates significant difference between the two follow-ups (subscript). Unit of all indices is *µ*m.

**Table 6:** The individual numbers for contrast sensitivity under photopic and scotopic conditions with or without glare at four spatial frequencies (*A*, *B*, *C*, and *D*).

	Photopic				Scotopic			
	*A*	*B*	*C*	*D*	*A*	*B*	*C*	*D*
Preoperative	1.58 ± 0.23	1.72 ± 0.14	1.37 ± 0.23	0.85 ± 0.21	1.53 ± 0.22	1.29 ± 0.37	0.59 ± 0.32	0.15 ± 0.34
6 month	1.56 ± 0.14	1.75 ± 0.51	1.32 ± 0.22	0.91 ± 0.23	1.50 ± 0.20	1.23 ± 0.30	0.59 ± 0.30	0.14 ± 0.34
30 month	1.48 ± 0.16	1.66 ± 0.20	1.34 ± 0.26	0.88 ± 0.28	1.41 ± 0.20	1.22 ± 0.30	0.67 ± 0.23	0.13 ± 0.33

	Photopic and Glare				Scotopic and Glare			
	*A*	*B*	*C*	*D*	*A*	*B*	*C*	*D*

Preoperative	1.70 ± 0.17	1.82 ± 0.15	1.42 ± 0.17	0.87 ± 0.23	1.54 ± 0.28	1.17 ± 0.36	0.53 ± 0.30	0.15 ± 0.34
6 month	1.62 ± 0.15	1.73 ± 0.17	1.29 ± 0.22	0.89 ± 0.19	1.46 ± 0.22	1.20 ± 0.33	0.46 ± 0.21	0.11 ± 0.28
30 month	1.54 ± 0.13	1.66 ± 0.27	1.34 ± 0.28	0.94 ± 0.33	1.40 ± 0.29	1.08 ± 0.34	0.46 ± 0.23	0.10 ± 0.35
	*P*_30‐b<0.001_	*P*_30‐b=0.015_						

*A*, *B*, *C*, and *D* mean 3, 6, 12, and 18 cycles/degree, respectively. Only *P* value <0.05 is shown in the table and indicates significant difference between the two follow-ups (subscript).

**Table 7:** The pupil size preoperatively and at 30 months postoperatively.

	Preop.	30-month	*P* value
Photopic (mm)	3.27 ± 0.55	3.10 ± 0.66	**0.030**
	2.44 to 4.45	2.32 to 4.48	

Scotopic (mm)	6.67 ± 0.52 mm	6.70 ± 0.78 mm	0.889
	**5.08 to 7.84**	**6.14 to 7.38**	

0.030 is the *p* value of the comparison of the photopic pupil diameter between the preoperative and the 30‐month postoperative ones. 5.08 to 7.84 is the range of the preoperative scotopic diameter. 6.14 to 7.38 is the range of the 30‐month postoperative scotopic diameter.

**Table 8:** The relationship between the changes of photopic pupil size and the changes of contrast sensitivity.

	Item	*β*	*P* value	95% CI	
				Lower limits	Upper limits
Photopic condition	*A*	0.031	0.884	−0.233	0.269
	*B*	−0.012	0.947	−0.221	0.207
	*C*	0.077	0.636	−0.222	0.356
	*D*	0.077	0.458	−0.273	0.428

Photopic and glare	*A*	−0.016	0.946	−0.231	0.216
	*B*	−0.261	0.115	−0.433	0.051
	*C*	0.282	**0.038**	0.016	0.504
	*D*	−0.179	0.293	−0.570	0.180

Scotopic	*A*	−0.331	0.077	−0.621	0.034
	*B*	−0.061	0.744	−0.569	0.412
	*C*	0.061	0.753	−0.361	0.492
	*D*	−0.098	0.623	−0.474	0.290

Scotopic and glare	*A*	−0.279	0.171	−0.724	0.136
	*B*	0.031	0.835	−0.318	0.390
	*C*	−0.013	0.936	−0.324	0.300
	*D*	−0.141	0.482	−0.19	0.291

*A*, *B*, *C*, and *D* mean 3, 6, 12, and 18 cycles/degree, respectively. The bold value “0.038” means to be statistically significant.

## Data Availability

The data used to support the findings of this study are available from the corresponding author upon request.
